# Lessons from climate modeling on the design and use of ensembles for crop modeling

**DOI:** 10.1007/s10584-016-1803-1

**Published:** 2016-09-15

**Authors:** Daniel Wallach, Linda O. Mearns, Alex C. Ruane, Reimund P. Rötter, Senthold Asseng

**Affiliations:** 1 INRA, UMR AGIR, Castanet Tolosan, France; 2 National Center for Atmospheric Research, Boulder, CO USA; 3 National Aeronautics and Space Agency Goddard Institute for Space Studies, New York, NY USA; 4 Georg-August-Universität Göttingen, Göttingen, Germany; 5 University of Florida, Gainesville, FL USA

**Keywords:** Model ensembles, Crop models, Climate models, Model weighting, Super ensembles

## Abstract

Working with ensembles of crop models is a recent but important development in crop modeling which promises to lead to better uncertainty estimates for model projections and predictions, better predictions using the ensemble mean or median, and closer collaboration within the modeling community. There are numerous open questions about the best way to create and analyze such ensembles. Much can be learned from the field of climate modeling, given its much longer experience with ensembles. We draw on that experience to identify questions and make propositions that should help make ensemble modeling with crop models more rigorous and informative. The propositions include defining criteria for acceptance of models in a crop MME, exploring criteria for evaluating the degree of relatedness of models in a MME, studying the effect of number of models in the ensemble, development of a statistical model of model sampling, creation of a repository for MME results, studies of possible differential weighting of models in an ensemble, creation of single model ensembles based on sampling from the uncertainty distribution of parameter values or inputs specifically oriented toward uncertainty estimation, the creation of super ensembles that sample more than one source of uncertainty, the analysis of super ensemble results to obtain information on total uncertainty and the separate contributions of different sources of uncertainty and finally further investigation of the use of the multi-model mean or median as a predictor.

**Electronic supplementary material:**

The online version of this article (doi:10.1007/s10584-016-1803-1) contains supplementary material, which is available to authorized users.

## Introduction

In modeling complex systems, it is important to have a measure of uncertainty in simulated values (Tebaldi and Knutti 2007). A promising way to evaluate this uncertainty is through working with ensembles of models; the variability among the models in the ensemble is a measure of our uncertainty as to how to model the system. Ensembles allow one to obtain a probability distribution instead of a point prediction (Harris et al. 2010). Furthermore, it has been empirically observed in many fields that ensemble averages or medians often better reproduce observations than even the best individual model (Hagedorn et al. 2005; Tebaldi and Knutti 2007; Palosuo et al. 2011; Martre et al. 2015). Additional benefits from working with ensembles of models arise from the closer collaboration between modeling groups.

Climate modeling and crop modeling are two fields in which multiple groups have developed different models to represent the same complex system. In both fields there is major interest in the uncertainty of simulations and the potential of ensemble statistics to improve predictions or projections. (As used here, “projection” is a potential future evolution of some quantity (e.g., future temperature), given assumptions about the future state of the world, while “prediction” (or forecast) is usually a definite statement or statistical estimate of an expected occurrence of an event in the future. Projections are generally more uncertain than predictions). It is therefore not surprising that both fields have major programs related to model intercomparison and the construction of multi-model ensembles (MMEs). However, the climate modeling community began working with MMEs with the Atmospheric Model Intercomparison project in 1989 (Gates et al. 1999), whereas global collaboration to create crop multi-model ensembles began in 2011 with the Agricultural Modeling Intercomparison and Improvement project (Rosenzweig et al. 2013), though more limited intercomparison studies predated that project (e.g., Jamieson et al. 1998; Mearns et al. 1999). Progress in the use of ensembles of both climate and crop models in studies of climate change is discussed in (Challinor et al. 2013). More background material on climate models and crop models is presented in supplementary material.

Many of the methods of working with ensembles, and many of the problems that arise, are common to the fields of climate and process-based crop modeling. Knutti (2010) explicitly suggests that some of the recommendations for working with ensembles of climate models could apply to other types of numerical models. Given the longer experience of the climate modeling community, it seems worthwhile to examine how their experience with ensemble modeling could contribute to crop modeling.

The objective of this paper is to identify questions and approaches related to developing and using model ensembles that have been studied in the climate modeling literature and are relevant to crop models. This should hopefully accelerate the progression of the crop model community in taking advantage of this useful diagnostic approach.

## Construction of model ensembles

The basic idea behind an ensemble is to carry out simulations using multiple models and/or multiple variants of a single model, with an aim to examine the uncertainty associated with a modeling exercise. In general model structure, model parameters and model inputs are all uncertain, and the ensemble can be created to represent the uncertainty in any of those, either singly or in combination.

### Choosing the participants in a multi-model ensemble (MME)

The choice of models clearly affects any conclusions based on a MME. A major question then is what models to include, or more generally what are the criteria for inclusion. (Knutti 2010).

To date, most climate model ensembles are “ensembles of opportunity” (Tebaldi and Knutti 2007). Any modeling group that wishes to participate can do so, assuming the model under consideration has an accepted set of model components. One problem is that some of the candidate models may be very poor determinants for hindcasts, and including them in the ensemble may unrealistically inflate the uncertainty estimations (Knutti 2010). However, an a priori determination of which models are poor is a very complex and difficult task, since this will largely depend on the particular metrics used, the variables being evaluated and the regions being investigated. It is also possible that all the models in an ensemble are ‘wrong’ in so far as they are missing important components of the climate system (e.g., submodels of glaciers). In fact we do not know how to thoroughly evaluate climate models for the sake of eliminating poor model performers (IPCC 2013a). However, the recently launched CMIP6 will require some verifiable test runs to demonstrate model quality (Meehl et al. 2014).

The relatively short experience with ensembles of crop models has also involved “ensembles of opportunity”. The need for quality criteria to exclude poorly performing models has been discussed (Palosuo et al. 2011; Rötter et al. 2012). Asseng et al. (2013) removed those models with the highest and lowest 10 % of simulated values in some of their analyses, but do not discuss that choice. Eliminating poorly performing models may be more important for crop than for climate MMEs, since in general crop models are less thoroughly evaluated. For example, Tubiello and Ewert (2002) found that the models most used for analyzing the effect of increased CO_2_ are those that have been evaluated the least using enhanced CO_2_ experiments, although this situation has evolved in the interim.

Following the example of the climate modeling community, it seems worthwhile to propose standardized tests for candidate models of crop MMEs. These tests should allow comparison with observations. Going further, it would be of interest to propose and test guidelines, based on standardized tests, for including (or excluding) models in crop MME studies.

### Evaluating the degree of relatedness of the models in a MME

Including closely related models in an ensemble brings the risk of giving undue weight to a single basic modeling approach. A first problem is determining how models are related. One approach with crop models has been to identify models that have similar equations for underlying processes, such as photosynthesis. In general, however, it has not been found that structural similarity leads to similarity in simulated values in crop MMEs (Palosuo et al. 2011; Martre et al. 2015; Li et al. 2015). An alternative approach, proposed for climate models (Bishop and Abramowitz 2013), is to examine the covariance in model errors as the measure of model dependence. High correlation of the model errors indicates that the simulations are not independent. This approach should be explored for crop models, in order to see what insights it brings into the structure of a MME.

### Determining the required number of models in a MME

The number of models in a crop MME is important, because it will affect both the mean and the variability of the ensemble outputs. It has practical implications because it is difficult to organize studies with multiple models. If it is sufficient to have fewer models, then such studies will be that much easier to conduct.

In crop modeling, emphasis has been on how the mean or median of an output variable of interest varies as the number of models is reduced, and in particular on the number of models required for the mean or median to stabilize (Asseng et al. 2013). However, this does not directly address the question of how many ensemble members are necessary to provide a satisfactory estimate of model uncertainty. This should be a topic of further study.

### Proposing a statistical sampling model for model ensembles

In order to better understand ensemble properties, and in particular in order to examine theoretically the effect of number of models in a MME, hypotheses about the population of models being sampled and the sampling process are necessary. One simple hypothesis proposed for climate models is the “truth plus error” paradigm, which assumes that the population of model predictions is correct on the average, but each model has some error drawn from a distribution of errors. A different hypothesis is the “indistinguishable” paradigm, which assumes that both model predictions and truth are drawn from the same distribution (Bishop and Abramowitz 2013). This topic has not been addressed in the crop modeling community. It is important to do so in order to provide a theoretical underpinning for discussion of MME properties. The hypotheses proposed for climate models provide a useful starting point.

### Creating model repositories

Given the effort involved in creating crop MME simulation studies, and their potential usefulness, it is of interest to create data repositories to make the results fully available to the community of researchers and end-users, as is done for climate models (Williams et al. 2011). As the climate modeling experience has shown, this would also spur research into the methodology and practice of using ensembles. The amount of data to be stored is significantly less for crop models than for climate models. On the other hand, a certain fragmentation of the work in crop modeling (for example for different crops) means that it may be harder to create one overall data repository. In any case, the example of the climate modeling community will be very valuable here.

### Assigning different weights to each model in a multi-model ensemble

Model weighting involves giving possibly different weights to results from different models in a MME. The objective is to improve estimates of uncertainty and/or to improve projections or predictions based on the ensemble mean or median.

In climate modeling, there have been a number of approaches testing alternative means of combining models, and this remains an active area of research. Knutti et al. (2010) identified important elements in studies concerning ranking or weighting of models. Among these, is the importance of explicitly explaining both the metric used for ranking and any statistical hypotheses about the models in the ensemble as a sample from a population of models. This clearly applies to crop MMEs as well.

Bayesian model averaging is particularly attractive, as a standard statistical method of taking into account model uncertainty (Wintle et al. 2003; Clyde and George 2004). In this approach one starts from a priori weights for each model (often equal weights), and then updates the weights based on model agreement with observations. This could improve both uncertainty estimation and prediction. As an example, Robertson et al. (2004) found that their Bayesian optimal weighting scheme for seasonal climate prediction outperformed the ensembles with equal weighting.

Giorgi and Mearns (2002) defined a reliability ensemble average (REA) where the weight assigned to each model depends both on that model’s agreement with observed data and its agreement with the ensemble average for projections. This approach has been used in multiple examinations of both global and regional climate model results (Sobolowski and Pavelsky 2012), and as a point of departure for formulating probability density functions (Tebaldi and Knutti 2007). More recently the method has been revised to dispense with the model convergence criterion (Xu et al. 2010), which has been controversial in some quarters.

Several different means of weighting different regional climate models in an ensemble have been suggested by Liu et al. (2010). They compare three weighting methods. The first is equal weights (the simple ensemble). The second weights by the inverse of fractional contribution to squared absolute error. The third method, found to be the best, calculates weights to minimize squared error of the final weighted ensemble.

A relatively recent approach in climate modeling is to weight models based both on performance and on between-model correlations of residual errors (e.g., Sanderson et al. 2015). Weighting based on correlations is supposed to remove much of the dependence between models, and thus make the sample more like a sample of independent models. Studies have shown that such a weighting scheme is superior to simple model averaging, with respect to both evaluating uncertainty and improving predictions (Bishop and Abramowitz 2013; Evans et al. 2013).

Model weighting however is not standard procedure in climate modeling. In the most recent Intergovernmental Panel on Climate Change (IPCC) report, it is acknowledged that the climate community does not know how to weight models to determine the best estimate of future climate change (Flato et al. 2013), though there is an example in that report where models are selected based on a verification protocol for projection of Arctic Sea Ice decrease throughout the twenty-first century.

As far as we know there has been no published work to date on differential weighting of crop models in a MME, although the question has been raised (Martre et al. 2015). Since crop model ensembles are often evaluated using quite limited amounts of data, it might seem that performance weighting of crop models is unlikely to be useful. On the other hand, a major argument against weighting of climate models based on hindcast error is that it assumes that the response to future radiative forcing will be consistent with that in the historical period, which cannot be tested. Crop models on the other hand can at least be tested against field experiments that impose higher atmospheric CO_2_ concentrations (Kimball et al. 1995; Ewert et al. 2002) and higher temperatures (Wall et al. 2011) than are observed today.

It would be of interest to explore weighting schemes for crop models, starting with the various approaches tested with climate models. A major decision in performance weighting is the choice of outputs to be considered. The choice is very large for climate models, but more constrained for crop models. Even here, however, it will be necessary to study whether it is preferable to base performance weighting just on the outputs of major interest (often just yield), or to use a larger group of output variables. For example, phenology is essentially always simulated, so part of weighting could be based on agreement with phenology data.

### Creating ensembles based on a single model with multiple parameter vectors

We can create a distribution of outcomes from a single model, by sampling from the probability distribution of uncertain quantities that affect the model outputs. This results in an ensemble of different model configurations, which are effectively different models though all use the same basic equations and structure. For climate models, the two sources of uncertainty within a single model that have been explored are uncertainties in parameter values and uncertainties in the initial conditions.

Ensembles which use the same model but multiple parameter values are referred to as “perturbed physics ensembles” or ‘parameter permutation experiments’ (PPEs). An example is described in Murphy et al. (2004) who estimated climate model uncertainty based on a 53-member ensemble of model versions using different parameter values. The uncertainty ranges of the parameters were determined by expert opinion, and the acceptability of the parameters was based on objective goodness-of-fit criteria for the model with those parameters. Other PPEs are described in (Yokohata et al. 2011) and (Sanderson 2011). It can, however, be difficult to quantify the uncertainty in the parameter values, in particular when these are ad hoc values without any clear theoretical or observational basis. Furthermore, while it is conceptually easy (one uses Monte Carlo sampling) to generate a distribution of outputs by sampling from the distributions of many or all of the parameters, it can be computationally very demanding. The use of model emulators can facilitate the evaluation of a broad number of parameter combinations (Murphy et al. 2007).

Although they are not generally called ensemble studies, there have been many studies where the effect of parameter uncertainty on the predictions of a particular crop model has been studied, including specifically in the study of the consequences of CO_2_ enrichment (Challinor and Wheeler 2008). Much of this work has been oriented toward sensitivity analysis (ranking parameters according to their effect on model outputs) rather than toward uncertainty estimates. As for climate models, a major difficulty is quantifying the uncertainty in the parameters. Most commonly, parameter uncertainty is based on the range of values found in the literature (Aggarwal 1995; Richter et al. 2010), and only a fairly small fraction of the total number of parameters is treated as uncertain. In these studies the evaluation of parameter vectors, to eliminate vectors that give unrealistic results, is not usually practiced. This could be considered in future studies.

There have also been a few parameter uncertainty estimates based on a Bayesian approach to model calibration (Iizumi et al. 2009; Wallach et al. 2012)). A simpler alternative that has been applied to crop models is the GLUE algorithm, which explores the space of possible parameter values, calculates a likelihood and eliminates parameter vectors whose likelihood is below a threshold (Wang et al. 2005). Controversy concerning GLUE due to its subjective aspects is summarized in (Beven and Binley 2014).

There is a need to pursue studies that quantify the contribution of parameter uncertainty to crop model prediction uncertainty. This will require improved approaches to quantifying parameter uncertainty. It will be useful to distinguish between those parameters estimated by calibration using data common to all the models in a MME, and the other model parameters based on data specific to each model.

### Creating ensembles based on a single model with multiple input values

Another type of climate model ensemble based on a single model results from varying the initial conditions that are used to start the simulations (e.g. Deser et al. 2012; Deser et al. 2014), thus exploring the internal variability that results. For example, Deser et al. (2014) examined the ensemble of results of the NCAR CCSM3 climate model with each member beginning from a slightly different initial atmospheric state.

For crop models, typical input variables are initial conditions, daily weather, soil properties and crop management. Here initial conditions have no special importance; many of the input variables are difficult to estimate and may have quite large uncertainties. Though not usually referred to as ensemble studies, there have been multiple studies of the effect of input uncertainty on crop model simulations (Bouman 1994; Aggarwal 1995; Moeller et al. 2009; Roux et al. 2014). Two specific types of input uncertainty have received particular attention recently. First, one method of upscaling crop model outputs from field to region or beyond is to execute a model in every grid cell, using a representative field for each cell (Rosenzweig et al. 2014). The uncertainty in the choice of representative field is in fact uncertainty in the input variables. Uncertainty due to scale change is explicitly studied in (Zhao et al. 2015). Secondly, in impact assessment studies, the uncertainty in the climate projections must be taken into account. This has led to running crop models with multiple future climates (some recent examples are Asseng et al. 2013; Li et al. 2015).

One analogy between climate and crop model inputs may be instructive. It has been argued that when studying the impact of climate change on crops and soil, it is important to do long-term simulations without reinitializing soil conditions each year (Basso et al. 2015). It would be worthwhile to investigate whether the uncertainty due to uncertain initial conditions increases with time, as in climate models.

More information concerning the importance of uncertainty in explanatory variables for crop models, in both absolute terms and relative to other sources of uncertainty, would be valuable.

### Super ensembles

The core concept of super ensembles is combining different types of ensembles, for example, MMEs and multiple initial conditions or multiple global climate models coupled with multiple regional climate models (Kendon et al. 2010). In the context of seasonal climate forecasts a common combination is multi-model experiments along with single model initial conditions realizations (Robertson et al. 2004). There have not been, to this point, efforts to combine climate models that represent the three different types of ensembles.

In crop modeling, there have been few studies that combine multiple sources of uncertainty. These include multiple crop models with multiple climate models (Mearns et al. 1999; Asseng et al. 2013; Li et al. 2015), multiple crop and climate models with multiple parameters (Tao et al. 2009) and multiple parameter values with multiple values for inputs (Aggarwal 1995).

Given that computational considerations are less limiting for crop models than for climate models, it would probably be feasible to do full factorial simulation experiments for crop models (multiple models, multiple inputs, multiple parameterizations for each model). It would be important to consider all of these sources of uncertainty in a common framework, in order to obtain better estimates of overall uncertainty and the relative importance of the different contributions to uncertainty.

## Analyzing the results of ensembles

### Quantifying and displaying uncertainty

The major motivation for working with ensembles is the information provided about uncertainty. Many of the difficulties of assessing and reporting uncertainty are described in (Wesselink et al. 2015).

Examining the variability within a multi-model ensemble (MME) is the central approach in IPCC reports to quantifying uncertainty in climate projections due to structural uncertainties. Results concern different output variables at various spatial scales. An atlas in the most recent report displays results for different scenarios of radiative forcing (IPCC 2013b), showing the 25th, 50th, and 75th percentiles of the model results.

Primarily Bayesian probabilistic methods have been used in quantifying uncertainty in PPEs whether they are used for quantifying uncertainty in climate sensitivity (Murphy et al. 2004) or in regional climate changes (Murphy et al. 2007). In examining a 57 member ensemble of PPEs, (Murphy et al. 2014) used fans of uncertainty (10th to 90th percentiles) to communicate uncertainty. In contrast, in analysis of the results of initial condition experiments, more analytic displays of uncertainty have been used. For example, (Deser et al. 2014) display individual results for the suite of initial condition simulations performed with the NCAR CCSM3.

As for climate models, crop MME studies have focused on inter-model variability to quantify uncertainty. In considering the effect of changed conditions, the uncertainty information is presented as an inter-model coefficient of variation (standard deviation of simulated values/mean simulated value), the inter-quartile range (Pirttioja et al. 2015) or as the percentage of models agreeing in the sign of change (Rosenzweig et al. 2014).

As discussed above, variability between models is not the only source of projection uncertainty. There is also parameter and input uncertainty to consider, as well as common biases that may cause the truth to fall outside of the ensemble spread. An important future activity in crop modeling will be to evaluate overall uncertainty and error, both for projections under uncertain climate and for predictions under testable conditions. Also, attention to the communication of uncertainty information is important.

An additional important topic that we do not explore in detail here is the role of data in determining estimations of uncertainty, whether it be the data used for model development, for calibration or the data used for comparison with simulated values.

### Evaluating the separate contributions to overall uncertainty

Another important step is to disaggregate total uncertainty among the various contributions, commonly referred to as sensitivity analysis (Saltelli et al. 2000). One approach that has been applied to climate models is that of analysis of variance, as suggested by Yip et al. (2011) and used in the context of regional and global climate models by Mearns et al. (2013). (Wallach et al. 2016) propose a random effects analysis of variance for crop model uncertainty, with analytical expressions for the contributions from model structure, parameters and inputs. (Asseng et al. 2013) use a simple comparison of variances to separate the contributions of multiple crop and multiple climate models to overall uncertainty. Further studies that implement these or other approaches for crop model simulation experiments are needed.

### Evaluating uncertainty estimates

An important question is the extent to which uncertainties estimated on the basis of one data set represent true uncertainty of new predictions or projections. If the uncertainty is represented by a probability distribution, then the true response for the new data should be within the estimated x% confidence intervals, x% of the time. This cannot be checked for climate projections, but could be checked for crop models under conditions such as increased CO_2_ and temperature, that partially imitate future climate.

### Using the ensemble average as estimator or predictor

Tebaldi and Knutti (2007) cite literature that shows that though the mean ensemble prediction may not be significantly better than the best model for each particular variable, the ensemble prediction usually does do better when performance over several variables is considered. However, the ensemble mean may not be best if common biases are present across all modeling groups or if a particular model features a unique aspect of crucial importance (Bukovsky et al. 2015).

Many MME studies with crop models have remarked that the mean or median of the simulated values seems to give good agreement with observed yields (Asseng et al. 2014; Bassu et al. 2014; Li et al. 2015; Palosuo et al. 2011). Martre et al. (2015) found that when multiple outputs are considered, the mean and median were both better predictors than even the best individual model, with the median being slightly better than the mean.

The conclusion that the mean or median is really a better estimator than the best model would imply that even without improving present-day models, one could obtain better predictions by using ensembles. Building off an expectation that ensemble medians are stronger predictors of future conditions than any individual model, Asseng et al. (2014) base extrapolations of future temperature impacts on world wheat production on the median of a MME. Given the potential importance of the mean or median in providing improved predictions, and the relatively limited supporting evidence to date, it seems important to test this hypothesis more thoroughly.

There has been only limited discussion of why the crop MME mean or median is a good predictor. In addition to the same arguments as for climate models one could add that different crop models are developed and tested using different data (except usually for a relatively small shared data set used for calibration) so in this sense crop model ensembles are based on more data than individual models (Martre et al. 2015). It is critical to better understand why the ensemble mean or median is a good predictor, as the basis for identifying the situations where this result will hold and where it will not.

Further exploration is also needed into how the choice of models, the number of models, and possible weighting of models affect the quality of the mean or median as a predictor. Once again, it is important to consider the output variables that are taken into account, since improvement may not concern all outputs equally.

## Conclusions

Working with ensembles of models is a recent, important development in crop modeling. The major advantage of doing so is that MMEs provide information on uncertainty in crop model estimations and projections related to model structure, which has been shown to be a major source of uncertainty. It has also been shown that the mean or median of multiple crop models can be a better estimator than even the best model. A third advantage of working with multi-model ensembles is the collaboration and exchanges that it fosters within the crop modeling community.

These important advantages, and the experience from the climate modeling community, strongly suggest that studies based on crop model ensembles may expand in the future to become a common way of working with crop models. Advancing the methodology of working with crop MMEs is necessary to realize the full potential of such studies.

This work will progress much faster to the extent that it builds on the large amount of research in this area already accomplished by the climate modeling community. Of course, given the differences between climate and crop modeling, not all of the lessons from climate modeling are applicable to crop models.

Table 1 summarizes the proposals that have been made here for ways forward for crop modeling based on ensembles. They are largely inspired by the work in the climate modeling community. The propositions include defining criteria for acceptance of models in a crop MME, exploring criteria for evaluating the degree of relatedness of models in a MME, studying the effect of number of models in the ensemble, development of a statistical model of model sampling, creation of a repository for MME results, studies of possible differential weighting of models in an ensemble, creation of single model ensembles based on sampling from the uncertainty distribution of parameter values or inputs specifically oriented toward uncertainty estimation, the creation of super ensembles that sample more than one source of uncertainty, the analysis of super ensemble results to obtain information on total uncertainty and the separate contributions of different sources of uncertainty and finally further investigation of the use of the multi-model mean or median as a predictor.

**Table 1:** Proposed actions to improve creation and use of crop MMEs and associated benefits

For details, see section	Proposed action (see section indicated for details)	Benefits
2.1	Develop standardized tests for candidate models of crop MMEs. Propose and test guidelines for including (or excluding) models in crop MME studies.	Clearer rules for creating crop MMEs, and better appreciation of consequences of those rules.
2.2	Test the use of covariances of errors as a way of quantifying the degree of relatedness of models in a MME	Improved insights into the structure of a MME. Basis for down-weighting related models.
2.3	Investigate the effect of number of models in a crop model MME on estimates of uncertainty and on the quality of the mean or median as a predictor	Information on minimum required number of models in a MME.
2.4	Develop and evaluate statistical assumptions about the sampling process underlying MMEs	Provide theoretical basis for studies of the properties of MMEs.
2.5	Create repositories for storing multi model results	Make crop MME results available for further studies.
2.6	Develop and test model weighting approaches for crop MMEs	Model weighting could improve estimate of uncertainty. Weighted mean could be better predictor than simple mean.
2.7	Carry out further studies of the uncertainty in crop model simulations due to parameter uncertainty. Develop improved methods of estimating parameter uncertainty	Evaluate importance of parameter uncertainty per se and in relation to other sources of uncertainty.
2.8	Carry out further studies of the uncertainty in crop simulations due to input uncertainty, including how uncertainty increases in multi-year simulations	Evaluate importance of input uncertainty per se and in relation to other sources of uncertainty.
2.9	Carry out simulation studies involving all of structure, parameter and input uncertainty	Create a common framework for estimating uncertainty from all of these sources of uncertainty.
3.1	Produce estimates of total uncertainty, including common biases of models	Produce information for evaluating overall confidence in simulated values.
3.2	Estimate the separate contributions to overall uncertainty	Prioritize future work on evaluating and possibly reducing uncertainty from some of the different sources.
3.3	Test whether ensemble based uncertainty estimates are realistic	Determine level of confidence in uncertainty estimates.
3.4	Carry out further studies on the mean or median of MMEs versus the best model. Develop a theoretical framework for evaluating the MME mean or median	Identify when the mean or median is likely to be a useful predictor.

## Electronic supplementary material

ESM 1 (DOCX 194 kb)
