# Supplementary material for: Lessons from climate modeling on the design and use of ensembles for crop modeling
Source: Clim Change. 2016 Sep 15;139(3):551–64. doi: 10.1007/s10584-016-1803-1 (PMC7175712; doi:10.1007/s10584-016-1803-1)
Supplement: Supplementary file 1 — (DOCX 194 kb) [file 10584_2016_1803_MOESM1_ESM.docx]

Lessons from climate modeling on the design and use of ensembles for crop modeling

Journal: Climatic Change

Daniel Wallach^1^, Linda O. Mearns^2^, Alex C. Ruane^3^, Reimund P. Rötter^4,5^,
and Senthold Asseng^6^

^1^INRA, UMR AGIR, Castanet Tolosan, France. wallach@toulouse.inra.fr

^2^National Center for Atmospheric Research, Boulder, Colorado, USA

^3^National Aeronautics and Space Agency Goddard Institute for Space Studies, New York, USA

^4^National Resources Institute Finland (LUKE), Helsinki, Finland

^5^University of Göttingen, Göttingen, Germany

^6^University of Florida, Gainesville, Florida, USA

Supplementary Material

# Introduction

The climate model community has been routinely working with ensembles of models for over two decades, dating back to the Atmospheric Model Intercomparison Project (AMIP) initiated in 1989 (Gates 1997), and comparisons of model simulations (i.e. small ensembles) date back at least to the first IPCC Report (Mitchell et al. 1990). Since then, ensemble studies have included not only multi-model ensembles but also combinations of multi-model ensembles with ensembles based on a single model. Figure 1 presents a schematic diagram of the different sorts of ensemble studies.

On the other hand, studies involving large crop model ensembles only date back a few years (Kersebaum et al. 2007; Palosuo et al. 2011; Rötter et al. 2012) . A first project that led to intercomparison of vegetation models was the Global Change and Terrestrial Ecosystems (GCTE) project of the International Geosphere-Biosphere Programme (IGBP), which led to the crop model intercomparison studies of (Jamieson et al. 1998; Supit et al. 2012). The first project comparable to AMIP to work with crop model ensembles, the Agricultural Model Intercomparison and Improvement Project (AgMIP) (Rosenzweig et al. 2013), began in 2010 and has already produced important crop model ensemble results (Asseng et al. 2013; Rosenzweig et al. 2014; Bassu et al. 2014; Asseng et al. 2014; Li et al. 2015). More recently the European project MACSUR (Ewert et al. 2015) has also applied large crop model ensembles, e.g. for crop-climate sensitivity analysis (Pirttioja et al. 2015) or in crop rotation modeling (Kollas et al. 2015).

# Brief description of climate models

Climate models are complex 3-dimensional mathematical representations of the physical, biological, and chemical processes that make up the climate system. These include: processes in the atmosphere, ocean, ice, vegetation, and land-surface as well as the interactions among these different parts of the system (Flato et al. 2013). Models that represent the entire globe are referred to as general circulation models or global climate models (GCMs), while those that model only a part of the globe are referred to as regional climate models (RCMs) (Rummukainen 2010; Rummukainen 2016). RCMs are driven by boundary conditions provided by global models, and are usually used to provide greater spatial detail for a particular region. Conventionally, global models that include at least a closed carbon cycle are referred to as Earth System Models (ESMs). Climate models are used for a range of purposes. The most fundamental is to develop greater understanding of the fully coupled climate system. The use most relevant to our purpose in this article is to investigate the impacts of anthropogenic climate change, or the response of the climate system to radiative forcing from changes in atmospheric greenhouse gases (GHGs) and aerosols as a result of human actions.

The origins of general circulation models of the atmosphere can be traced back to around 1955 with a climate conference in Princeton, published as *Proceedings of a Conference on the Application of Numerical Integration Techniques to the Problem of the General Circulation*. Soon thereafter various research groups initiated general circulation modeling activities of the atmosphere and oceans (Edwards 2000). The development of climate system models mostly started with atmospheric models in the 1960s. Work on ocean circulation models also was under development at this time. The further development of the models from the 1960s to now reflects improvements in how different processes were modeled but also the inclusion of more and more processes. Into the 1970s and 80s the modeling of sea ice was added to that of the atmosphere and ocean. By the 1990s land surface models included vegetation models, and the atmospheric models included the effect of sulfate aerosols, as well as solar forcing from aerosols.

In the past ten years there has been a rise in the inclusion of complete carbon cycles (which conventionally divides the GCMs from the Earth System Models; ESMs), and more complete biogeochemical cycles have been added (Flato et al. 2013). As these improvements have been made, the resolution at which these calculations have been performed has increased considerably, from 5 to 7 degrees latitude and longitude to about 1 degree for long term (a century and longer) simulations. The added complexity and improved spatial resolution has gone hand in hand with increased computational capacity of large computers. Regional climate models developed from regional weather prediction models principally in the 1990s (Dickenson et al. 1989; Giorgi 1990) and allowed greater spatial and temporal detail by concentrating computational power on a smaller region at finer resolution.

The results of climate model simulations that consider the effect of changing GHGs and aerosols have been used to investigate what the impacts of climate change on human and natural systems may be since the 1980s. Use of climate model simulation results as input for crop models was indeed one of the earliest uses (Terjung et al. 1984; Santer 1985; Liverman et al. 1986; Parry and Carter 1989), but such results are now used in all important impacts sectors (e.g., water resources, forestry, natural ecosystems, human health). Precipitation and temperature (on different spatial and temporal scales) are the most common variables that are used from the simulations, but humidity, wind speed and solar radiation are also used, for example, as input for crop models for simulating evapotranspiration.

Results from the climate models are typically not used directly owing to biases common at coarse resolution. In the first couple of decades changes in climate (future – current) were calculated and then these changes were imposed upon observations – the so-called delta approach (Mearns et al. 2001). While there remain sizable errors in climate model output there are now a greater range of methods for “bias correction” or “bias adjustment” of climate model data, such as the bias correction spatial disaggregation (BCSD) method of (Maurer et al. 2007). A comparison of methods is given in (Räty et al. 2014). These same procedures can be used to correct biases in both global and regional model simulations, although these approaches have their strengths and weaknesses and are the basis of ongoing research (Mearns et al. 2014).

# Brief description of crop models

When we refer to crop models here, we refer exclusively to process-based crop simulation models. Such models translate the process understanding about how a crop grows in interaction with the environment and as a result of management practices into mathematical algorithms. Typical input data for a crop model are daily weather information such as solar radiation, maximum and minimum temperatures, relative humidity and rainfall, soil characteristics, initial soil conditions, cultivar characteristics, and crop management information. Using such data, the models simulate the dynamics of crop growth and development including phenology, biomass accumulation (either total or of separate compartments), leaf area formation, yield and uptake of water and nutrients. Soil processes involving water, nitrogen, and carbon are also typically simulated, as well as associated stresses when plant needs are not met.

Uses of crop models include: formalizing our understanding of the dynamics of the soil-plant-atmosphere system; simulation and comparison of the effectiveness of crop management strategies; and assessment of the impacts of climate variability and change in different environmental settings. Much recent work has concentrated on the impacts of climate change and on *ex ante* analysis of various adaptation strategies to climate change at the crop or cropping system level.

Crop simulation modelling began in the 1960s when the first mathematical relationships were developed between biomass growth and solar radiation (Wit 1959; Monteith 1965), In successive decades a number of different modelling teams around the world have taken these equations and integrated them with representations of other important crop physiological processes as well as crop management measures into process-based crop models e.g. (Weir et al. 1984; Jones et al. 2003; Keating et al. 2003; Stöckle et al. 2003; van Ittersum et al. 2003; Brisson et al. 2006). An example of the diversity of crop models is the 30 models that participated in the AgMIP Wheat Team’s intercomparison of extreme heat response (Asseng et al. 2014).

While initially complex crop models with very high temporal and spatial resolution were developed (Bouman et al. 1996), most crop models now operate with a daily time step on the scale of a field, assumed to have identical properties and inputs over its entire area. Early crop model applications on assessing climate change impacts took place at the field scale. Later, frameworks were constructed to run crop models on grids covering larger areas such as regions, nations, large watersheds or globally by using climate or environmental stratifications in conjunction with (representative) point simulations (Supit et al. 2012; Angulo et al. 2013; Boogaard et al. 2013; Rosenzweig et al. 2014). Large-area crop models have also been developed to match the scales of climate model grid boxes (Challinor et al. 2004; Tao et al. 2009a; Tao et al. 2009b).

# Differences between climate and crop models

There are of course many differences between climate and crop models. We emphasize here several differences that are important with respect to the construction and interpretation of model ensembles.

Perhaps the major difference concerns the treatment of space. Global climate models simulate the entire surface of the earth, the three dimensional ocean, and the three dimensional atmosphere. Regional climate models, as stated earlier, cover a portion of the earth’s surface, usually on the order of continental or subcontinental scales, and also include the three dimensions of the atmosphere. In contrast, the majority of crop models used today are single point models (with results presented on a per-hectare basis) more akin to column radiation models still used in the climate community. Gridded crop models that cover much larger geographic areas are typically single models that are applied using input data organized spatially over a grid. Simulations are conducted independently for each grid box, with no horizontal interaction among locations. Large-area crop models represent aggregated conditions over a wider area but lose adherence to a specific, observable field.

A second important difference is in the multiplicity of modeled systems. All global coupled models are modeling the same system, namely the earth’s climate system, although they may vary regarding which subsystems are more completely modeled. On the other hand, there are different (though often related) crop models for each crop species. Thus many of the activities related to ensembles of models – preparing the ensembles and analyzing the results – need to be repeated multiple times in the realm of crop modeling. For example, there have already been separate multi-model ensemble studies of potato (Kabat 1995), barley (Rötter et al. 2012), wheat (Palosuo et al. 2011; Asseng et al. 2013), maize (Bassu et al. 2014) and rice (Li et al. 2015) Depending on the crop, the number of models and the data available for testing can vary considerably.

A third major difference is in the amount and types of observational data needed and available for model calibration and validation. For climate modeling, large amounts of some types of data are readily available, including daily weather variables (especially temperature, precipitation, and surface pressure) at many sites around the globe for the last several decades. These data networks have dense coverage in parts of the world, however others remain data sparse (e.g., Arctic and Antarctic regions, over the oceans, boreal zones of Canada and Siberia, and the subtropical areas of many developing countries). Also, since so many different variables are simulated by climate models, the availability of observations for all the atmospheric variables (e.g., wind, pressure, humidity, radiation, aerosols) remains problematic, particularly for upper levels of the atmosphere. Data on other components of the climate system (e.g., ocean temperatures and salinity, glacial and sea ice, and vegetation characteristics) are difficult to observe at the scales required for global modeling. It is a reasonable assumption that all climate models have access to the same data.

Crop models are generally calibrated and evaluated based on a relatively small number of cropping seasons, though usually with a range of treatments and in various climate environments. Furthermore, agronomic experiments are usually designed to examine crop response to some specific crop or soil management factor and not to calibrate and validate a crop model; as a consequence several important input and output variables for one or more of the sub-systems interacting in the real world (as well as in the model) are commonly neglected – not measured at all or with insufficient accuracy (Kersebaum et al. 2015). Another important factor limiting data availability is that there is as yet no standardized way to share data internationally. Thus, different models are commonly developed and calibrated using different data sets. The differences in calibration data can have an important effect on model results (Angulo et al. 2013; Craufurd et al. 2013). Finally, results are often crop variety specific, and varieties change over time and over space. The limited data availability also means that there is much less standardized data for evaluation of crop models. However, it also must be recognized that considerably fewer variables are simulated in crop models compared to climate models, as discussed below, and in that regard, the data requirements for calibration and validation are lower if we consider a single crop.

A fourth major difference is in the number of variables, as well as spatial and temporal scales, that are of analyzed in climate modeling. These include average temperatures or extreme temperatures, average rainfall, humidity, winds, and radiation, 500 mb geopotential heights, ocean temperature and salinity at different depths, and many others (Gleckler et al. 2008). In crop modeling the focus is often limited to yield on a field level or regional basis, with either seasonal or long-term averages of primary interest. Other variables that may also be of interest include crop phenology, water use, above-ground biomass, harvest index, grain protein content, soil nutrient balances and nutrient losses. In addition many other variables are calculated, but these mainly help in diagnosing the cause for any changes in yield or as the focus of non-climate studies (e.g., runoff management, farm sustainability, or precision agriculture applications).

Another difference is in the potential for model evaluation. We can test climate models using hindcasts, but no experimentation imitating future conditions is possible. We will only know how well a model performs for mid-century by waiting until that time. We can assume that skill in hindcasts reflects skill in projecting future climate, but of course that may not be true. On the other hand, we can test crop models against field experiments with temperatures higher than those currently experienced at a particular location by using spatial analogs. Crop models can also be tested against field experiments that artificially imposed higher atmospheric CO_2_ concentrations (Kimball et al. 1995; Ewert et al. 2002) and higher temperatures (Wall et al. 2011) than are observed today. Thus, we have the possibility of evaluating crop models for conditions that at least mimic some aspects of putative future conditions. However, the range of future conditions, and in particular combinations of future conditions, that have been tested is limited.

Finally, the computational resources required for crop and climate models are quite different. Computation time can be a strong limiting factor in climate modeling, and even with current super computers it is difficult if not impossible to explore large numbers of models each with multiple parameterizations and input conditions. On the other hand, it is currently quite feasible to do millions of simulations with most advanced crop models. The amount of data generated in each case is also quite different. Climate models typically generate terabytes of data, whereas crop models, with many fewer output variables, generate much less output data.

# Crop models embedded within climate models

Many global climate models (or the more advanced Earth System Models, Heavens et al. 2013) essentially now incorporate crop models in their land surface schemes. Some of these models are both used off-line and incorporated into the land surface scheme for fully coupled simulations. For the most part the purpose of incorporating such crop models into the land surface schemes is to improve the reproduction of land surface characteristics in agricultural regions, but they now are also being used to simulate crop yields for their own sake (for example ORCHIDEE (Krinner et al. 2005).

# REFERENCES

Angulo C, Rötter R, Lock R, et al (2013) Implication of crop model calibration strategies for assessing regional impacts of climate change in Europe. Agric For Meteorol 170:32–46. doi: 10.1016/j.agrformet.2012.11.017

Asseng S, Ewert F, Martre P, et al (2014) Rising temperatures reduce global wheat production. Nat Clim Chang 5:143–147. doi: 10.1038/nclimate2470

Asseng S, Ewert F, Rosenzweig C, et al (2013) Uncertainty in simulating wheat yields under climate change. Nat Clim Chang 3:827–832. doi: 10.1038/nclimate1916

Bassu S, Brisson N, Durand J-L, et al (2014) How do various maize crop models vary in their responses to climate change factors? Glob Chang Biol 20:2301–20. doi: 10.1111/gcb.12520

Boogaard H, Wolf J, Supit I, et al (2013) A regional implementation of WOFOST for calculating yield gaps of autumn-sown wheat across the European Union. F Crop Res 143:130–142. doi: 10.1016/j.fcr.2012.11.005

Bouman BAM, van Keulen H, van Laar HH, Rabbinge R (1996) The “School of de Wit” crop growth simulation models: A pedigree and historical overview. Agric Syst 52:171–198. doi: 10.1016/0308-521X(96)00011-X

Brisson N, Wery J, Boote KJ (2006) Fundamental concepts of crop models illustrated by a comparative approach. In: D. W, Makowski D, Jones JW (eds) Working with dynamic crop models. Evaluation, analysis, parameterization and applications. Elsevier, Amsterdam, pp 257–279

Challinor AJ, Wheeler TR, Craufurd PQ, et al (2004) Design and optimisation of a large-area process-based model for annual crops. Agric For Meteorol 124:99–120.

Craufurd PQ, Vadez V, Jagadish SVK, et al (2013) Crop science experiments designed to inform crop modeling. Agric For Meteorol 170:8–18. doi: 10.1016/j.agrformet.2011.09.003

Dickenson RE, Errico RM, Giorgi F, Bates G (1989) A regional climate model for the Western United States. Clim Change 15:383–422.

Edwards PN (2000) A Brief History of Atmospheric General Circulation Modeling. In: Randall DA (ed) General Ci. Academic Press, New York,

Ewert F, Rodriguez D, Jamieson P, et al (2002) Effects of elevated CO2 and drought on wheat: testing crop simulation models for different experimental and climatic conditions. Agric Ecosyst Environ 93:249–266. doi: 10.1016/S0167-8809(01)00352-8

Ewert F, Rötter RP, Bindi M, et al (2015) Crop modelling for integrated assessment of risk to food production from climate change. Environ Model Softw 72:287–303. doi: 10.1016/j.envsoft.2014.12.003

Flato G, Marotzke J, Abiodun B, et al (2013) Evaluation of Climate Models. In: Stocker T., Qin D, Plattner G-K, et al. (eds) Climate Change 2013: The Physical Science Basis. Contribution of Working Group I to the Fifth Assessment Report of the Intergovernmental Panel on Climate Change. Cambride University Press, Cambridge United Kingdom and New York,

Gates WL (1997) AMIP: The Atmospheric Model Intercomparison Project. In: Progr. Clim. Model Diagnosis Intercomp. http://www-pcmdi.llnl.gov/publications/PCMDIrept7/AMIPexp.html. Accessed 27 Feb 2014

Giorgi F (1990) Simulation of regional climate using a limited area model nested in a general-circulation model. J Clim 3:941–963.

Gleckler PJ, Taylor KE, Doutriaux C (2008) Performance metrics for climate models. J Geophys Res 113:D06104. doi: 10.1029/2007JD008972

Heavens NG, Ward DS, Natalie MM (2013) Studying and projecting climate change with earth system models. Nat Educ Knowl 4(5):4.

Jamieson PD, Porter JR, Goudriaan J, et al (1998) A comparison of the models AFRCWHEAT2, CERES-Wheat, Sirius, SUCROS2 and SWHEAT with measurements from wheat grown under draught. F Crop Res 55 :23–44.

Jones JW, Hoogenboom G, Porter CH, et al (2003) The DSSAT cropping system model. Eur J Agron 18:235–265.

Kabat P (1995) Modelling and parameterization of the soil-plant-atmosphere system : a comparison of potato growth models. Wageningen Pers, Wageningen, The Netherlands

Keating B., Carberry P., Hammer G., et al (2003) An overview of APSIM, a model designed for farming systems simulation. Eur J Agron 18:267–288. doi: 10.1016/S1161-0301(02)00108-9

Kersebaum KC, Boote KJ, Jorgenson JS, et al (2015) Analysis and classification of data sets for calibration and validation of agro-ecosystem models. Environ Model Softw. doi: 10.1016/j.envsoft.2015.05.009

Kersebaum KC, Hecker JM, Mirschel W, Wegehenkel M (2007) Modelling water and nutrient dynamics in soil–crop systems: a comparison of simulation models applied on common data sets. In: Kersebaum KC, Hecker JM, Mirschel W, Wegehenkel M (eds) Modelling water and nutrient dynamics in soil–crop systems. Springer Netherlands, Dordrecht, pp 1–17

Kimball B, Pinter P, Garcia R, et al (1995) Productivity and water use of wheat under free-air CO2 enrichment. Glob Chang Biol 1:429–442. doi: 10.1111/j.1365-2486.1995.tb00041.x

Kollas C, Kersebaum KC, Nendel C, et al (2015) Crop rotation modelling—A European model intercomparison. Eur J Agron 70:98–111. doi: 10.1016/j.eja.2015.06.007

Krinner G, Viovy N, de Noblet-Ducoudré N, et al (2005) A dynamic global vegetation model for studies of the coupled atmosphere-biosphere system. Global Biogeochem Cycles 19:n/a–n/a. doi: 10.1029/2003GB002199

Li T, Hasegawa T, Yin X, et al (2015) Uncertainties in predicting rice yield by current crop models under a wide range of climatic conditions. Glob Chang Biol 21:1328–41. doi: 10.1111/gcb.12758

Liverman DM, Terjung WH, Hayes JT, Mearns LO (1986) Climatic change and grain corn yields in the North American Great Plains. Clim Change 9:327–347. doi: 10.1007/BF00139076

Maurer EP, Brekke L, Pruitt T, Duffy PB (2007) Fine-resolution climate projections enhance regional climate change impact studies. Eos Trans Am Geophys Union 88:504.

Mearns LO, Bukovsky M, Pryor S, Magana V (2014) Downscaling of climate information. In: Ohring G (ed) Climate change in North America, Springer. New York, pp 201–250

Mearns LO, Hulme M, Carter TR, et al (2001) Climate scenario development. In: Houghton JT et al. (ed) Climate Change 2001: The Scientific Basis. Contribution of Working Group 1 to the Third Assessment Report of the IPCC, Cambridge. Cambridge,

Mitchell JFB, Manabe S, Tokioka T, Meleshko V (1990) Equilibrium Climate Change. In: Houghton, J. G, Jenkins J, Ephraums JJ (eds) Climate Change: The IPCC Scientific Assessment, Cambride U. Cambridge,

Monteith JL (1965) Light Distribution and Photosynthesis in Field Crops. Ann Bot 29:17–37.

Palosuo T, Kersebaum KC, Angulo C, et al (2011) Simulation of winter wheat yield and its variability in different climates of Europe: A comparison of eight crop growth models. Eur J Agron 35:103–114. doi: 10.1016/j.eja.2011.05.001

Parry ML, Carter TR (1989) An assessment of the effects of climatic change on agriculture. Clim Change 15:95–116. doi: 10.1007/BF00138848

Pirttioja N, TR C, Fronzek S, et al (2015) Temperature and precipitation effects on wheat yield across a European transect: a crop model ensemble analysis using impact response surfaces . Clim Res 65:87–105.

Räty O, Räisänen J, Ylhäisi JS (2014) Evaluation of delta change and bias correction methods for future daily precipitation: intermodel cross-validation using ENSEMBLES simulations. Clim Dyn 42:2287–2303. doi: 10.1007/s00382-014-2130-8

Rosenzweig C, Elliott J, Deryng D, et al (2014) Assessing agricultural risks of climate change in the 21st century in a global gridded crop model intercomparison. Proc Natl Acad Sci U S A 111:3268–73. doi: 10.1073/pnas.1222463110

Rosenzweig C, Jones JW, Hatfield JL, et al (2013) The Agricultural Model Intercomparison and Improvement Project (AgMIP): Protocols and pilot studies. Agric For Meteorol 170:166–182. doi: 10.1016/j.agrformet.2012.09.011

Rötter RP, Palosuo T, Kersebaum KC, et al (2012) Simulation of spring barley yield in different climatic zones of Northern and Central Europe: A comparison of nine crop models. F Crop Res 133:23–36. doi: 10.1016/j.fcr.2012.03.016

Rummukainen M (2010) State-of-the-art with regional climate models. Wiley Interdiscip Rev Clim Chang 1:82–96. doi: 10.1002/wcc.8

Rummukainen M (2016) Added value in regional climate modeling. Wiley Interdiscip Rev Clim Chang 7:145–159. doi: 10.1002/wcc.378

Santer B (1985) The use of general circulation models in climate impact analysis ? A preliminary study of the impacts of a CO2- induced climatic change on West European agriculture. Clim Change 7:71–93. doi: 10.1007/BF00139442

Stöckle CO, Donatelli M, Nelson R (2003) CropSyst, a cropping systems simulation model. Eur J Agron 18:289–307. doi: 10.1016/S1161-0301(02)00109-0

Supit I, van Diepen CA, de Wit AJW, et al (2012) Assessing climate change effects on European crop yields using the Crop Growth Monitoring System and a weather generator. Agric For Meteorol 164:96–111. doi: 10.1016/j.agrformet.2012.05.005

Tao F, Yokozawa M, Zhang Z (2009a) Modelling the impacts of weather and climate variability on crop productivity over a large area: A new process-based model development, optimization, and uncertainties analysis. Agric For Meteorol 149:831–850. doi: 10.1016/j.agrformet.2008.11.004

Tao F, Zhang Z, Liu J, Yokozawa M (2009b) Modelling the impacts of weather and climate variability on crop productivity over a large area: A new super-ensemble-based probabilistic projection. Agric For Meteorol 149:1266–1278.

Terjung WH, Liverman DM, Hayes JT (1984) Climatic change and water requirements for grain corn in the North American Great Plains. Clim Change 6:193–220. doi: 10.1007/BF00144612

van Ittersum M., Leffelaar P., van Keulen H, et al (2003) On approaches and applications of the Wageningen crop models. Eur J Agron 18:201–234. doi: 10.1016/S1161-0301(02)00106-5

Wall GW, Kimball BA, White JW, Ottman MJ (2011) Gas exchange and water relations of spring wheat under full-season infrared warming. Glob Chang Biol 17:2113–2133. doi: 10.1111/j.1365-2486.2011.02399.x

Weir AH, Bragg PL, Porter JR, Rayner JH (1984) A winter wheat crop simulation model without water or nutrient limitations. J Agric Sci 102:371. doi: 10.1017/S0021859600042702

Wit CT de (1959) Potential photosynthesis of crop surfaces. Netherlands J Agric Sci 7:141–149.

Figure caption

Fig 1 Schematic diagram of various types of climate model ensembles

a). Multi model ensemble (MME). The simulations in this case come from n different models (model_1_,…,model_n_), each with a single parameter vector specific to that model (θ_1_ for for model_1_ etc) and all with the same initial conditions IC. The simulated output Y (Y_m1_ for the output from model_1_ etc) could be any variable or combination of variables.

b). Initial condition ensemble. The same model, with a specific parameter vector θ, is run for multiple initial conditions IC_1_,…,IC_n_. The simulated output Y (Y_IC1_ for the output using initial condition IC_1_, etc) could be any variable or combination of variables.

c). Perturbed parameter ensemble. The same model, with a specific set of input conditions IC, is run using multiple parameter vectors θ_1_,…,θ_n_. The simulated output Y (Y_θ1_ for the output using parameter vector θ_1_, etc) could be any variable or combination of variables.

d). Super ensemble combining parameter and model structure uncertainty. The simulations in this case come from n different models (model_1_,…,model_n_), each with multiple parameter vectors specific to that model (θ_m1_,_1_,…, θ_m1_,_p_ for model_1_ etc) and all with the same initial conditions IC. The simulated output Y (Y_m1,1_ for the output from model_1_ using parameter vector θ_m1_,_1_ etc) could be any variable or combination of variables.

1. Multi-model Ensemble

IC

θ_m2_

model_2_

Y_m1_

Y _m2_

Y _mn_

model_1_

model_n_

θ_m1_

θ_mn_

a

IC_1_

IC_2_

IC_n_

θ

model

Y_IC1_

Y_IC2_

Y_ICn_

b. Initial Condition (IC) Ensemble

model

model

IC

θ_2_

model

Y_θ1_

Y_θ2_

Y_θn_

θ_1_

θ_n_

c. Perturbed Parameter Ensemble

model

model

Y _mn,1_

d. Super ensemble combining parameter and model structure uncertainty

model_2_

model_1_

model_1_

model_1_

model_2_

model_2_

model_3_

model_3_

model_n_

θ_m2,1_

θ_mn,1_

θ_m1_,_1_

Y_m1,p1_

Y_m,p1_

Y_m1,1_

Y _m2,1_

Y _m2,1_

Y _m2,1_

Y _mn,1_

Y _mn,1_

IC
